# Investigation into repetitive concussion in sport (RECOS): study protocol of a prospective, exploratory, observational cohort study

**DOI:** 10.1136/bmjopen-2019-029883

**Published:** 2019-07-04

**Authors:** Kamal M Yakoub, David J Davies, Zhangjie Su, Conor Bentley, Mario Forcione, Emma Toman, Douglas Hammond, Callum N Watson, Jon Bishop, Lauren Cooper, Aron K Barbey, Vijay Sawlani, Valentina Di Pietro, Michael J Grey, Antonio Belli

**Affiliations:** 1 NIHR Surgical Reconstruction and Microbiology Research Centre, University Hospitals Birmingham NHS Foundation Trust, Birmingham, UK; 2 Neurotrauma and Ophthalmology Research Group, Institute of Inflammation and Aging, University of Birmingham, Birmingham, UK; 3 Head Injury Management Research Group, Faculty of Clinical and Biomedical Science, School of Dentistry, University of Central Lancashire, Preston, UK; 4 The Beckman Institute for Advanced Science andTechnology, University of Illinois at Urbana Champaign, Illinois, USA; 5 Department of Neuroradiology, Queen Elizabeth Hospital Birmingham, University Hospitals Birmingham NHS Foundation Trust, Birmingham, UK; 6 Acquired Brain Injury Rehabilitation Alliance(ABIRA), School of Health Sciences, University of East Anglia, Norwich, UK

**Keywords:** concussion, traumatic brain injury, sport concussion, biomarkers, imaging, postconcussion syndrome

## Abstract

**Introduction:**

Sport-related concussion management remains a diagnostic dilemma to clinicians in all strata of care, coaching staff and players alike. The lack of objective diagnostic and prognostic biomarkers and over-reliance on subjective clinical assessments carries a significant health risk of undiagnosed concussive episodes and early return to play before full recovery increasing the risk of sustaining additional concussion, and leading to long-term sequelae and/or unfavourable outcome.

**Objective:**

To identify a set of parameters (neuroimaging with neurophysiological, biological and neuropsychological tests) that may support pitch-side and outpatient clinical decision-making in order to objectively diagnose concussion, determine the severity of injury, guide a safe return to play and identify the potential predictors of the long-term sequelae of concussion.

**Methods and analysis:**

An exploratory, observational, prospective, cohort study recruiting between 2017 and 2020. The participants will have a baseline preseason screening (brain imaging, neuropsychological assessments, serum, urine and saliva sampling). If a screened player later suffers a concussion and/or multiple concussions then he/she will be assessed again with the same protocol within 72 hours, and their baseline data will be used as internal control as well as normative data. Inferential statistical analysis will be performed to determine correlations between biological, imaging techniques and neuropsychological assessments.

**Ethics and dissemination:**

This study was approved by the East of England—Essex Research Ethics Committee on 22 September 2017—REC 17/EE/0275; IRAS 216703. The results of this study will be presented at national and international conferences and submitted for publication in peer reviewed journals.

**Trial registration number:**

ISRCTN16974791; Pre-results.

**Strengths and limitations of this study**

- Prospectively recruiting from across the contact sporting continuum, this study will collect baseline and postconcussion biological samples with corresponding neuropsychological, neurophysiological and neuroimaging tests.
- The study will help to establish a multidisciplinary approach to objectively diagnose sport-related concussion and guide a safe return to play following single and repetitive concussions.
- To intercorrelate neuroimaging with neurophysiological, biological and neuropsychological tests for the prospective development of potential pitch-side and ambulatory technologies.
- This study will recruit from a population of adult athletes and therefore applications of study findings to other populations, such as non-athletes, will require further validation.
- Neuroimaging may not be suitable for all the participants, such as pregnant, claustrophobic athletes and those with metallic implants.

## Introduction

Sport-related concussion (SRC) is defined as a brief period of loss of consciousness, memory loss, feeling dazed or confused following trauma to the head, face or neck, and is a common cause of mild traumatic brain injury (mTBI).^1 2^ In the USA, the Centers for Disease Control and Prevention estimate that 1.6–3.8 million concussions occur in sports and recreational activities annually, but there is fear that the number may be much larger, as the majority of incidents are unrecognised or unreported.^3^

The majority of patients improve rapidly following a single concussion, but 10%–20% of individuals have persistent symptoms (eg, headache, dizziness, fogginess, imbalance and anxiety) at 3 months. In a minority (2%–4%) the symptoms may become permanent.^4 5^ There is a higher risk of sustaining further concussion if they returned to play before full recovery which prolongs recovery time after each incident.^4 5^

Certain groups of patients, such as athletes, soldiers and children are at greater risk of repetitive mTBI; potentially leading to a catastrophic form of brain injury known as second impact syndrome (SIS), thought to be due to the second insult occurring during a window of metabolic vulnerability of the brain.^6–8^

In susceptible individuals, repetitive head trauma has been linked to early neurodegenerative conditions such as Parkinson’s, amyotrophic lateral sclerosis and Alzheimer’s disease. The risk of neurodegenerative conditions has been demonstrated to treble in retired professional American football players^9^ and following the media attention dedicated to this issue, the link between mTBI and chronic traumatic encephalopathy (CTE) has become a major public concern.^10 11^ Compounding this problem is the fact that athletes, like soldiers, are highly trained, young, fit, motivated individuals proven to under-report concussive symptoms.^12 13^

Athletes are, therefore, at risk of SIS and CTE. Currently, there is no validated clinical biomarker available to assess mTBI, SIS and cumulative brain damage, leading to an over-reliance on self-reporting of symptoms in the management of mTBI.^14^

At present, the mainstream assessment of mTBI both in sports and in the primary/secondary healthcare setting involves the functional and symptomatic assessment of an individual using neurocognitive tests.^15^ These tests have significant limitations, particularly the lack of baseline/premorbid measurement in terms of sensitivity and specificity^16 17^ and the susceptibility to multiple confounding factors^18^ such as musculoskeletal injury and/or premorbid disability. This together with their requirement for a high level of engagement and concentration limits their application in outpatient clinic or pitch-side.

## Methods and analysis

### Study design

A cohort of contact sport athletes (eg, rugby, football, American football and so on) will be recruited throughout the West Midlands region and through referrals from sports clubs anywhere in the Great Britain.

The assessments are carried out in two settings: (1) the sports clubs with clinical areas dedicated to sample taking and data collection and (2) Birmingham concussion clinic at the Queen Elizabeth Hospital Birmingham (QEHB) or at the University of Birmingham (UoB).

Eligible individuals will be identified by key members of their coaching staff who have been trained regarding the inclusion specifications and the procedures involved in recruitment (table 1). Athletes who sustain a head injury during competition or training will be advised to attend an assessment session between 48 and 72 hours after their concussion at the concussion clinic at QEHB, although attending directly or as soon as is practically possible will be advised. Should the injury be sufficiently serious that the attending staff believes immediate medical assistance or transfer to the nearest emergency department is required, then standard clinical procedures should be followed in these cases.

**Table 1:** Summary of eligibility criteria

Inclusion criteria	Exclusion criteria
Male or female athletes participating in contact sports, aged 16–65 years, with fluent English speaking.	Individuals who require hospital admission after initial assessment for their TBI.
Single or repetitive mTBI sustained in contact sport <72 hours prior to assessment.	Intracranial haemorrhage, brain tissue injury or non-TBI related pathologies on initial CT/MRI scan.
Normal neurological objective examination at the time of enrolment.	Pregnancy (urine pregnancy test will be performed for confirmation).
	Any history of neurodegenerative pathology or any recent or ongoing illness affecting the central nervous system (eg, Parkinson’s, multiple sclerosis, meningitis, epilepsy, neoplasm).
	History of chronic alcoholism or drug abuse.
	Any other sustained injury that requires hospital admission.

mTBI, mild traumatic brain injury.

Consent to participate in the study will be obtained from the participants in a standardised written consent form. Details of the consent will be maintained as per the UoB and University Hospitals Birmingham NHS Foundation Trust guidelines, and maintained for the study period and archived thereafter.

Consented non-concussed athletes will participate in a baseline screening consisting of same assessments as for the concussed players, although these may not include MRI screening in all subjects due to resources constrains. The uninjured cohort provides normative data, as well as internal control data if a screened player later suffers a concussion during the season. Preseason screening assessments will be conducted at either the UoB site or the QEHB site or at the club facilities.

Up to 400 singly or multiply concussed athletes (where the second concussion has occurred within 21 days of the first event) will be recruited, consented and assessed by:

- Sampling of 25 mL venous blood, 2 mL saliva and 5 mL urine for metabolomics and genomic analysis (table 2).
- The immediate postconcussion assessment and cognitive testing is a computerised neurocognitive test for evaluating SRC. It measures multiple aspects of cognitive functioning, and consists of five testing composites, that is, verbal memory, visual memory, processing speed, reaction time and impulse control.^19^
- The screening module of neuropsychological assessment battery (Digit Span).^18^
- Wechsler Adult Intelligence Scale Version 4 (Digit Symbol Coding and Symbol Search).^20^
- Nine hole peg test to assess fine motor skills; the individual will be asked to place and remove nine pegs at one time, as quickly as possible, from nine holes in a peg board. This test evaluates coordination of upper limbs and hand dexterity.^21^
- The medical symptom validity test is a validated and computerised performance validity test. It is designed to assess the degree to which the participant has engaged appropriately in the testing process.^22^
- Balance assessments including virtual reality system, gait analysis, modified balance error scoring system (mBESS).^20–27^

**Table 2:** Analysis of biological fluids

Blood	Urine	Saliva
Metabolomics (NAA and related metabolites). Hormones (progesterone, aldosterone, 11-deoxycortisol, corticosterone, testosterone, androstenedione, cortisol, 17OHP, DHEA, DHEAS, cortisone). Pro- and anti-inflammatory cytokines (TNFα, IL1β, IL6, IL4, IL8, IL10, IL13, IL17, GM-CSF). Proteomics. Microparticles. Biomarkers of brain injury (S100B, GFAP and NSE). MicroRNAs.	Hormone profile. Brain biomarkers (NAA, S100B and GFAP). Metabolomics. MicroRNAs.	MicroRNAs. Metabolomics.

The mBESS test will be performed whereby a trained member of the repetitive concussion in sport team will assess the number of errors participants make while undertaking eyes closed dual, single leg and a tandem stances for 30 s, respectively.^27^

- An Immersion questionnaire to complete after the virtual reality system to investigate their level of immersion and sense of presence in the virtual reality environment and questionnaire using the metabolic equivalent of tasks will be used to assess participants’ physical activity levels prior to the sustaining of a concussion.^28^
- MRI to assess neuronal energy metabolism, functional brain network and tissue mechanical parameters.
- Functional near-infrared spectroscopy (fNIRS) to assess brain activation patterns during neurocognitive tasks.

**Figure 1:**
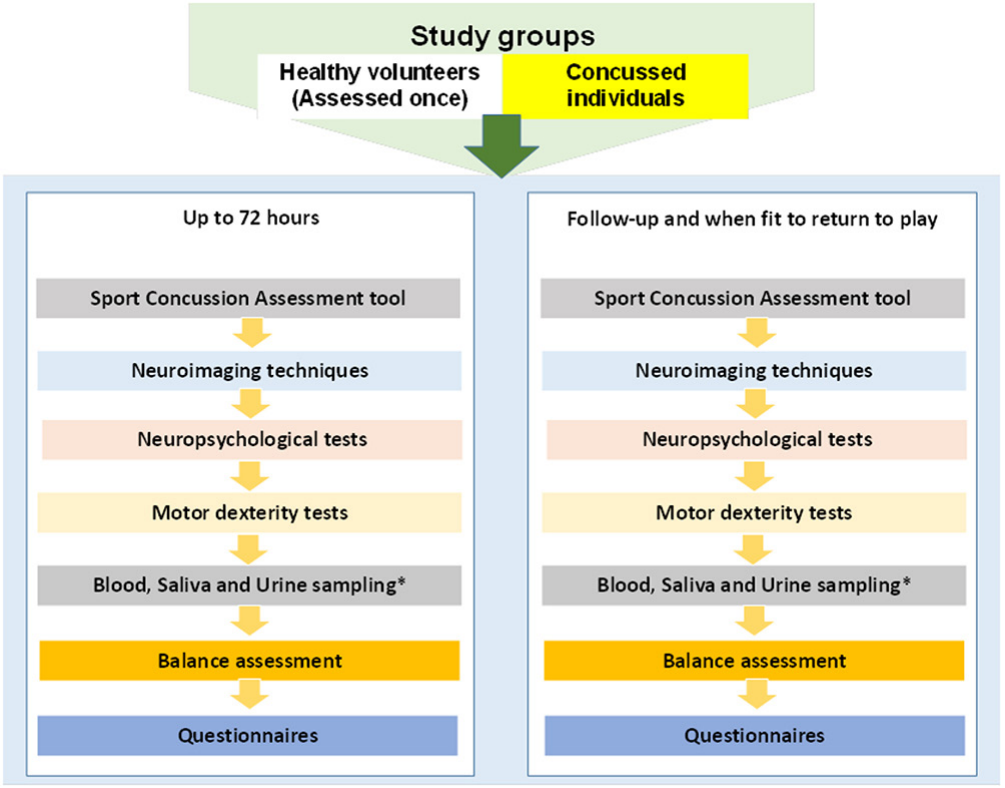
Flowchart of study procedures. ^*^Urine pregnancy test will be performed first for all female participants.

The Sport Concussion Assessment Tool—5th Edition^27^ may be taken within the sport club facilities immediately after concussion. Any other data will be collected during the pitch-side clinical assessment on concussed athletes within the Rugby Football Union guidelines, that is, the injury surveillance programme, may be used in the study. After the match the concussed participants will visit QEHB or the UoB or dedicated sport club facilities where all the assessments will be completed with short breaks in-between when necessary (figure 1).

MRI scanning (~1 hour) will be performed using a 3T MRI scanner at the QEHB. First of all, a standard structural MRI scan will be acquired (T1-weighted, T2-weighted and T2*-weighted and FLAIR images), followed by MR spectroscopy (1H-MRS) (NAA/choline, NAA/creatine and choline/creatine ratios),^29^ resting-state functional MRI (fMRI) (subjects are awake, eyes closed, motionless), diffusion tensor imaging (DTI) and magnetic resonance elastography (MRE) on brain will be performed to obtain quantitative values for tissue mechanical parameters.^30 31^

Prior to any female participant undergoing MRI, a pregnancy test will be performed after the expressed consent of the individual.

These assessments will be repeated in the QEHB or sports facilities during the follow-up and when the individual is deemed fit to return to play, until normalisation of the 1H-MRS or self-reported symptoms are observed.

The near-infrared spectroscopy (NIRS) is a safe non-invasive brain imaging technique that utilises near-infrared (NIR) light absorption to assess brain oxygen saturation. The NIR light is currently widely used in clinical practice (eg, pulse oximeter and brain monitoring during cardiac surgery). fNIRS measurements are performed once within 48–72 hours from the injury using a frequency domain NIRS device (ISS IMAGENT; Champaign, Illinois, USA) and a digitizer (POLHEMUS FASTRAK; Colchester, Vermont, USA).^32 33^

### Outcome measures and statistical analysis

The recruitment target is based on the size of the convenient sample that can be recruited (~400); power calculation do not apply as the investigation is explorative at this stage. The sample size will be open-ended and reviewed after 3 years.

Correlations between biomarkers, 1H-MRS, fMRI, MRE, DTI, fNIRS, neuropsychological scores, motor and coordination parameters will be assessed and reported with standard parametric statistical tests after normalisation of data or non-parametric test if normalisation were not possible. Time to resolution of symptoms will be compared for all the above modalities, as well as self-reported outcomes. Biochemical and imaging data will also be analysed with standard group comparison statistics (eg, t-test on normalised data or Mann-Whitney on non-normalisable data).

### Patient and public involvement

Links with sports academics and coaching staff have been established throughout the region through the University of Birmingham School of Sports, Exercise and Rehabilitation Sciences (SportexR) and through sports authorities such as the Rugby Football Union, the Welsh Rugby Union, GB Basketball, GB Hockey and the Football Association. Awareness of these programmes will be promoted to the potential cohort of athletes through event days organised at individual recruitment sites coordinated centrally through the UoB. In terms of dissemination, the study team will regularly update the clubs on the progress of the study and will report the findings by email/newsletter and in various meetings where club representatives are in attendance.

### Ethics and dissemination

All data will be collected and stored in accordance with the 1998 UK Data Protection Act, UoB and QEHB data handling and maintenance guidelines, with the minimum amount of required information recorded. The complied and analysed results will be presented at national and international conferences. Results will also be submitted for peer review and publication in the subject journals/literature.

### Data statement

All analysed data arising from the study will be kept on NHS and UoB servers. The computers on this network have restricted physical access; data are stored under coded filenames and the local network has secure password access restricted to a limited set of people. The raw MRI data are kept on a separate DICOM server on another network behind its own firewall which is only accessible by a small number of system administrators. Data spreadsheets will be kept on computers with password protected access and coded filenames, the original paper files will be secured in locked filing cabinets.

The datasets generated and/or analysed during the current study are/will be available on request from, Professor Antonio Belli, who is the point of contact and will be able to provide anonymised data with sufficient details to able to reproduce the analyses for up to 10 years.

## Supplementary Material

Reviewer comments

Author's manuscript
