# Supplementary material for: Investigation into repetitive concussion in sport (RECOS): study protocol of a prospective, exploratory, observational cohort study
Source: BMJ Open. 2019 Jul 4;9(7):e029883. doi: 10.1136/bmjopen-2019-029883 (PMC6615833; doi:10.1136/bmjopen-2019-029883)
Supplement: Author's manuscript [file bmjopen-2019-029883.draft_revisions.pdf]

BMJ Open is committed to open peer review. As part of this commitment we make the peer review history of every article we publish publicly available.

When an article is published we post the peer reviewers' comments and the authors' responses online. We also post the versions of the paper that were used during peer review. These are the versions that the peer review comments apply to.

The versions of the paper that follow are the versions that were submitted during the peer review process. They are not the versions of record or the final published versions. They should not be cited or distributed as the published version of this manuscript.

BMJ Open is an open access journal and the full, final, typeset and author-corrected version of record of the manuscript is available on our site with no access controls, subscription charges or pay-per-view fees (http://bmjopen.bmj.com).

If you have any questions on BMJ Open's open peer review process please email info.bmjopen@bmj.com

BMJ Open

# An investigation into Repetitive Concussion in Sport (RECOS): A study protocol of a prospective, observational cohort study

| | |
|---|---|
| Journal: | *BMJ Open* |
| Manuscript ID | bmjopen-2019-029883 |
| Article Type: | Protocol |
| Date Submitted by the Author: | 15-Feb-2019 |
| Complete List of Authors: | Yakoub, Kamal; NIHR SRMRC, Trauma<br>Davies, David; NIHR Surgical Reconstruction and Microbiology Research Centre, University Hospitals Birmingham NHS Foundation Trust<br>Su, Zhangjie; NIHR SRMRC<br>Bentley, Conor; NIHR Surgical Reconstruction and Microbiology Research Centre, University Hospitals Birmingham NHS Foundation Trust<br>Forcione, Mario; NIHR Surgical Reconstruction and Microbiology Research Centre, University Hospitals Birmingham NHS Foundation Trust,<br>Toman, Emma; NIHR Surgical Reconstruction and Microbiology Research Centre, Neurosurgery<br>Hammond, Douglas; Head Injury Management Research Group, Faculty of Clinical and Biomedical Science, School of Dentistry, University of Central Lancashire,<br>Watson, Callum; Neurotrauma and Ophthalmology Research Group, Institute of Inflammation and Aging, University of Birmingham,<br>Bishop, Jon; NIHR Surgical Reconstruction and Microbiology Research Centre, Medical Statistician<br>Cooper, Lauren; NIHR Surgical Reconstruction and Microbiology Research Centre, University Hospitals Birmingham NHS Foundation Trust Queen Elizabeth Hospital Birmingham<br>Barbey, Aron; The Beckman Institute for Advanced Science and Technology, University of Illinois<br>Sawlani, Vijay; University Hospitals Birmingham NHS Foundation Trust, Department of Radiology, Queen Elizabeth Hospital,<br>Di Pietro, V; University of Birmingham,<br>Grey, Michael<br>belli, antonio; University of Birmingham, Institute of Inflammation and Ageing |
| Keywords: | Concussion, Traumatic brain injury, Sport concussion, Biomarkers, Imaging, Post-concussion syndrome |
| | |

SCHOLARONE™
Manuscripts

# Title

An investigation into Repetitive Concussion in Sport (RECOS): A study protocol of a prospective, observational cohort study

# Authors

Kamal M Yakoub[1,2], David J Davies[1,2], Zhangjie Su[1], Conor Bentley[1,2], Mario Forcione[1,2] , Emma Toman[3], Douglas Hammond[3], Callum N Watson[2], Jonathan R B Bishop[1], Lauren Cooper[1], Aron K Barbey[6], Vijay Sawlani[1,4], Valentina Di Pietro[1,2,6], Michael Grey[5], Antonio Belli[1,2]

# Correspondence to

Dr Kamal M Yakoub

NIHR Surgical Reconstruction and Microbiology Research Centre

University Hospitals Birmingham NHS Foundation Trust

4th Floor West, Institute of Translational Medicine

Heritage Building (Queen Elizabeth Hospital)

B15 2TH

Birmingham

United Kingdom

E-mail: k.yakoub@bham.ac.uk

Telephone: +44(0)1213718223

# Author affiliations

1 NIHR Surgical Reconstruction and Microbiology Research Centre, University Hospitals Birmingham NHS Foundation Trust, Birmingham, UK

2 Neurotrauma and Ophthalmology Research Group, Institute of Inflammation and Aging, University of Birmingham, Birmingham, UK

3 Head Injury Management Research Group, Faculty of Clinical and Biomedical Science, School of Dentistry, University of Central Lancashire, Preston, UK

4 Department of Neuroradiology, Queen Elizabeth Hospital Birmingham, University Hospitals Birmingham NHS Foundation Trust, Birmingham, UK

5 Acquired Brain Injury Rehabilitation Alliance (ABIRA), School of Health Sciences, University of East Anglia, Norfolk, UK

6 The Beckman Institute for Advanced Science and Technology, University of Illinois at Urbana Champaign, Illinois, USA



# Keywords

Concussion; Traumatic brain injury; Sport concussion; Biomarkers; Imaging; Post-concussion syndrome

# Abstract

## Introduction

Sport-related concussion (SRC) management remains a diagnostic dilemma to clinicians in all strata of care, coaching staff and players alike. The lack of objective diagnostic and prognostic biomarkers and over-reliance on subjective clinical assessments carries a significant health risk of undiagnosed concussive episodes and early return to play before full recovery increasing the risk of sustaining additional concussion, and leading to long-term sequelae and/or unfavorable outcome.

## Objective

To identify a set of parameters (neuroimaging with neurophysiological, biological and neuropsychological tests) that may support pitch-side and outpatient clinical decision making in order to objectively diagnose concussion, determine the severity of injury, guide a safe return to play and identify the potential predictors of the long-term sequelae of concussion.

## Methods and analysis

An observational, prospective, cohort study recruiting between 2017 and 2020. The participants will have a baseline pre-season screening (brain imaging, neuropsychological assessments, serum, urine and saliva sampling). If a screened player later suffers a concussion and/or multiple concussions then he/she will be assessed again with the same protocol within 72hours, and their baseline data will be used as internal control as well as normative data. Inferential statistical analysis will be performed to determine correlations between biological, imaging techniques and neuropsychological assessments.

For peer review only

### Ethics and dissemination

Ethics approval has been obtained. The results of this study will be presented at national and international conferences and submitted for publication in peer reviewed journals.

## Article summary

### Strengths and limitations of this study

- This study will contribute to better understanding of the extent and nature of the metabolic and physiological window of vulnerability present in cerebral tissue following mild traumatic brain injury (mTBI) sustained during sport.
- The study will help to determine the relationship between a single concussion and subsequent/consecutive episodes in terms of their effect on that window of brain vulnerability with a view to guiding return to play following single and repetitive concussions.
- To inter-correlate neuroimaging with neurophysiological, biological and neuropsychological tests for the prospective development of potential pitch-side and ambulatory technologies.
- This study will recruit from a population of adult athletes and therefore applications of study findings to other populations, such as non-athletes, will require further validation.
- Neuroimaging may not be suitable for all the participants, such as pregnant, claustrophobic athletes, and those with metallic implants.

For peer review only

# Introduction

Sport-related concussion (SRC) is defined as a brief period of loss of consciousness (LOC), memory loss or feeling dazed or confused following trauma to the head, face or neck, and is a common cause of mild traumatic brain injury (mTBI).[1,2] In the US, the Centers for Disease Control and Prevention estimate that 1.6 to 3.8 million concussions occur in sports and recreational activities annually, but there is fear that the number may be much larger, as the majority of incidents are unrecognized or unreported.[3]

The majority of patients improve rapidly following a single concussion, but 10-20% of individuals have persistent symptoms (e.g. headache, dizziness, fogginess, imbalance, and anxiety) at three months. In a minority (2-4%) the symptoms may become permanent. [4,5] There is a higher risk of sustaining further concussion if they returned to play before full recovery which prolongs recovery time after each incident.[4, 5]

Certain groups of patients, such as athletes, soldiers and children are at greater risk of repetitive mild traumatic brain injury (mTBI); potentially leading to a catastrophic form of brain injury known as second impact syndrome (SIS), thought to be due to the second insult occurring during a window of metabolic vulnerability of the brain.[6-8]

In susceptible individuals, repetitive head trauma has been linked to early neurodegenerative conditions such as Parkinson's, amyotrophic lateral sclerosis and Alzheimer's disease. The risk of neurodegenerative conditions has been demonstrated to treble in retired professional American football players [9] and following the media attention dedicated to this issue, the link between mTBI and chronic traumatic encephalopathy (CTE) has become a major public concern. [10, 11]

Compounding this problem is the fact that athletes, like soldiers, are highly trained, young, fit, motivated individuals proven to under-report concussive symptoms.[12, 13]

Athletes are, therefore, at risk of SIS and CTE. Currently, there is no validated clinical biomarker available to assess mTBI, SIS and cumulative brain damage, leading to an over-reliance on self-reporting of symptoms in the management of mTBI.[14]

At present, the mainstream assessment of mTBI both in sports and in the primary/secondary healthcare setting involves the functional and symptomatic assessment of an individual using neurocognitive tests.[15] These tests have significant limitations, particularly the lack of baseline/premorbid measurement in terms of sensitivity and specificity [16, 17] and the susceptibility to multiple confounding factors [18] such as musculoskeletal injury and/or pre-morbid disability. This together with their requirement for a high level of engagement and concentration limits their application in outpatient clinic or pitch-side.

## Methods and analysis

### Study design

A cohort of contact sport athletes (e.g. rugby, football, American football, etc.) will be recruited throughout the West Midlands region and through referrals from sports clubs anywhere in the Great Britain.

The assessments are carried out in two settings, (1) the sports clubs with clinical areas dedicated to sample taking and data collection and (2) Birmingham concussion clinic at the Queen Elizabeth Hospital Birmingham (QEHB) or at the University of Birmingham (UoB).

For peer review only

Eligible individuals will be identified by key members of their coaching staff who have been trained regarding the inclusion specifications and the procedures involved in recruitment (Table 1). Athletes who sustain a head injury during competition or training will be advised to attend an assessment session between 48h and 72h after their concussion at the concussion clinic at QEHB, although attending directly or as soon as is practically possible will be advised. Should the injury be sufficiently serious that the attending staff believes immediate medical assistance or transfer to the nearest Emergency Department is required, then standard clinical procedures should be followed in these cases.

Consent to participate in the study will be obtained from the participants in a standardised written consent form. Details of the consent will be maintained as per the UoB and University Hospitals Birmingham NHS Foundation Trust (UHBNFT) guidelines, and maintained for the study period and archived thereafter.

Consented non-concussed athletes will participate in a baseline screening consisting of same assessments as for the concussed players, although these may not include Magnetic Resonance Imaging (MRI) screening in all subjects due to resources constrains. The uninjured cohort provides normative data, as well as internal control data if a screened player later suffers a concussion during the season.  Pre-season screening assessments will be conducted at either the University of Birmingham site or the QEHB site or at the club facilities.

For peer review only

**Table 1. Summary of eligibility criteria**

| Inclusion criteria | Exclusion criteria |
|---|---|
| Male or female athletes participating in contact sports, aged 16-65 years, with fluent English speaking. | Individuals who require hospital admission after initial assessment for their TBI. |
| Single or repetitive mTBI sustained in contact sport less than 72 hours prior to assessment. | Intracranial haemorrhage, brain tissue injury, or non-TBI related pathologies on initial CT/MRI scan. |
| Normal neurological objective examination at the time of enrolment. | Pregnancy (urine pregnancy test will be performed for confirmation). |
| | Any history of neurodegenerative pathology or any recent or ongoing illness affecting the central nervous system (e.g. Parkinson's, multiple sclerosis, meningitis, epilepsy, neoplasm). |
| | History of chronic alcoholism or drug abuse. |
| | Any other sustained injury that requires hospital admission. |

Up to 400 singly or multiply concussed athletes (where the second concussion has occurred within 21 days of the first event) will be recruited, consented and assessed by:

- Sampling of 25ml venous blood, 2ml saliva and 5ml urine for metabolomics and genomic analysis (Table 2).
- The Immediate post-concussion assessment and cognitive testing (ImPACT) is a computerised neurocognitive test for evaluating sport-related concussion. It measures multiple aspects of cognitive functioning, and

For peer review only

consists of five testing composites, i.e. verbal memory, visual memory, processing speed, reaction time, and impulse control.[19]

- The Screening Module of Neuropsychological Assessment Battery (NAB) (Digit Span).[18]
- Wechsler Adult Intelligence Scale Version 4 (WAIS-IV) (Digit Symbol Coding and Symbol Search).[20]
- Nine Hole Peg Test (NHPT) to assess fine motor skill; the individual will be asked to place and remove nine pegs at one time, as quickly as possible, from nine holes in a peg board. This test evaluates coordination of upper limbs and hand dexterity. [21]
- The Medical Symptom Validity Test (MSVT) is a validated and computerized performance validity test. It is designed to assess the degree to which the participant has engaged appropriately in the testing process. [22]
- Balance assessments (Virtual Reality-Wii Balance Board system, gait analysis, Modified Balance Error Scoring System (mBESS)).
  Individuals stand on a Wii Balance Board to measure centre of pressure changes whilst being perturbed in a virtual reality environment. Sufficient safety measures will be put in place to ensure participants do not fall from the raised platform. Gait analysis will be also undertaken. [20-27]
  A mBESS test will also be performed whereby a trained member of the RECOS team will assess the number of errors participants make whilst undertaking eyes closed dual, single leg and a tandem stances for 30 seconds respectively.[27]
- An Immersion questionnaire to complete after the virtual reality system to investigate their level of immersion and sense of presence in the virtual reality environment and questionnaire using the Metabolic Equivalent of

Tasks (MET) will be used to assess participants' physical activity levels prior to the sustaining of a concussion.[28]

- MRI to assess neuronal energy metabolism, functional brain network and tissue mechanical parameters.
- Functional Near-Infrared Spectroscopy (fNIRS) to assess brain activation patterns during neurocognitive tasks.

The Sport Concussion Assessment Tool – 5th Edition (SCAT5)[27] may be taken within the sport club facilities immediately after concussion. Any other data will be collected during the pitch-side clinical assessment on concussed athletes within the Rugby Football Union guidelines, i.e. the Injury Surveillance Programme, may be used in the study. After the match the concussed participants will visit QEHB or the UoB or dedicated sport club facilities where all the assessments will be completed with short breaks in-between when necessary (figure 1).

Magnetic Resonance (MR) scanning (approximately 1h) will be performed using a 3T MR scanner at the QEHB. First of all, a standard structural MR scan will be acquired (T1-, T2-, and T2* -weighted and FLAIR images), followed by MR Spectroscopy (1H-MRS) (NAA/Choline, NAA/Creatine and Choline/Creatine ratios.)[29], resting-state functional MRI (fMRI) (subjects are awake, eyes closed, motionless), Diffusion tensor imaging (DTI), and Magnetic Resonance Elastography (MRE) on brain will be performed to obtain quantitative values for tissue mechanical parameters.[30, 31]

Prior to any female participant undergoing MR imaging, a pregnancy test will be performed after the expressed consent of the individual.

These assessments will be repeated in the QEHB or sports facilities during the follow up and when the individual is deemed fit to return to play, until

For peer review only - http://bmjopen.bmj.com/site/about/guidelines.xhtml

normalisation of the MR Spectroscopy (1H-MRS) or self-reported symptoms are observed.

The Near-Infrared Spectroscopy (NIRS) is a safe non-invasive brain imaging technique that utilises Near-Infrared (NIR) light absorption to assess brain oxygen saturation. The NIR light is currently widely used in clinical practice (e.g. pulse oximeter and brain monitoring during cardiac surgery). fNIRS measurements are performed once within 48-72 hours from the injury using a frequency domain NIRS device (ISS™ IMAGENT; Champaign, Illinois, USA) and a digitizer (POLHEMUS FASTRAK®; Colchester, Vermont, USA).[32,33]

**Table 2. Analysis of biological fluids**

| Blood | Urine | Saliva |
|---|---|---|
| 1. Metabolomics (NAA and related metabolites)<br>2. Hormones (Progesterone, Aldosterone,11-deoxycortisol, Corticosterone, Testosterone, Androstenedione, Cortisol, 17OHP, DHEA, DHEAS, Cortisone)<br>3. Pro and anti-inflammatory cytokines (TNFα, IL1β, IL6, IL4, IL8, IL10, IL13, IL17, GM-CSF)<br>4. Proteomics<br>5. Microparticles<br>6. Biomarkers of brain injury (S100B, GFAP and NSE) | 1. Hormone profile<br>2. Brain biomarkers (NAA, S100B and GFAP)<br>3. Metabolomics<br>4. MicroRNAs | 1. MicroRNAs<br>2. Metabolomics |

For peer review only

| | | |
|---|---|---|
| 7. MicroRNAs | | |

For peer review only

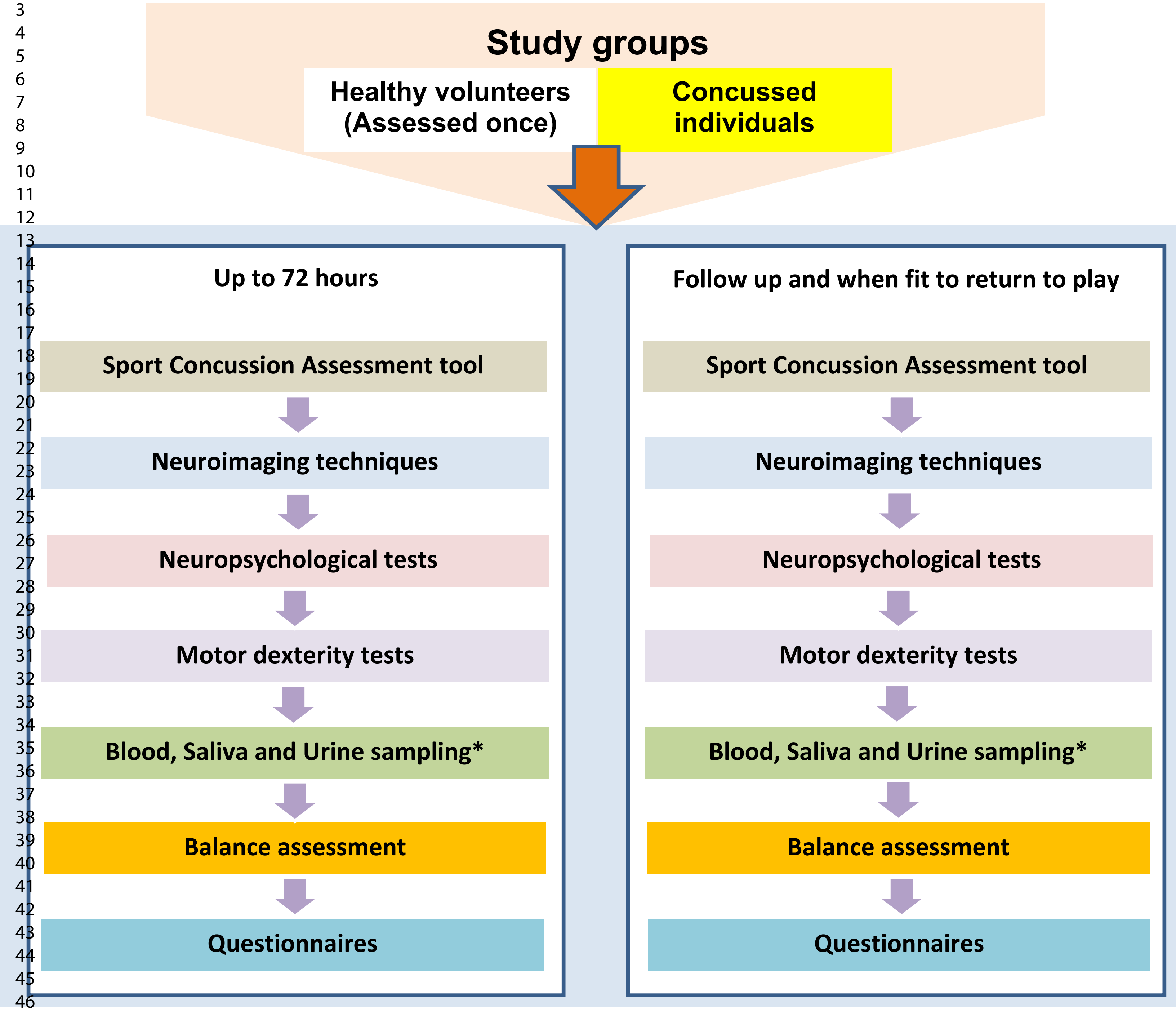


**Figure 1: Flowchart of study procedures. *urine pregnancy test will be performed first for all female participants**

## Outcome measures and statistical analysis

The recruitment target is based on the size of the convenient sample that can be recruited (approximately 400); power calculation do not apply as the investigation is explorative at this stage. The sample size will be open-ended and reviewed after 3 years.

Correlations between biomarkers, 1H-MRS, fMRI, MRE, DTI, fNIRS, neuropsychological scores, motor and coordination parameters will be assessed and reported with standard parametric statistical tests after normalisation of data or non-parametric test if normalisation were not possible. Time to resolution of symptoms will be compared for all the above modalities, as well as self-reported outcomes. Biochemical and imaging data will also be analysed with standard group comparison statistics (e.g. t-test on normalised data or Mann-Whitney on non-normalisable data).

## Patient and Public Involvement

Links with sports academics and coaching staff have been established throughout the region through the University of Birmingham School of Sports, Exercise and Rehabilitation Sciences (SportexR) and through sports authorities such as the Rugby Football Union, the Welsh Rugby Union, GB Basketball, GB Hockey and the Football Association. Awareness of these programs will be promoted to the potential cohort of athletes through event days organised at individual recruitment sites coordinated centrally through the UoB. In terms of dissemination, the study team will regularly update the clubs on the progress of the study and will report the findings by email/newsletter and in various meetings where club representatives are in attendance.

For peer review only - http://bmjopen.bmj.com/site/about/guidelines.xhtml

# Ethics and dissemination

This study was approved by the East of England - Essex Research Ethics Committee on 22 September 2017 – REC 17/EE/0275; IRAS 216703.

ISRCTN, ISRCTN16974791. Registered on 16/05/2018.

All data will be collected and stored in accordance with the 1998 UK Data Protection Act, UoB and QEHB data handling and maintenance guidelines, with the minimum amount of required information recorded. The complied and analysed results will be presented at national and international conferences. Results will also be submitted for peer review and publication in the subject journals/literature.

## Data statement

All analysed data arising from the study will be kept on NHS and UoB servers. The computers on this network have restricted physical access; data are stored under coded filenames and the local network has secure password access restricted to a limited set of people. The raw MRI data are kept on a separate DICOM server on another network behind its own firewall which is only accessible by a small number of system administrators. Subject score sheet data will be kept on computers with password protected access and coded filenames, the original paper files will be secured in locked filing cabinets.

The datasets generated and/or analysed during the current study are/will be available upon request from, Professor Antonio Belli, who is the point of contact and will be able to provide anonymised data with sufficient detail to able to reproduce the analyses for up to 10 years.

For peer review only

## Acknowledgements

The authors acknowledge UoB, NIHR SRMRC and British Medical Association for their support. Also, we would like to thank all the sports authorities, clubs and academics that agreed to participate in the study.

The research was funded by the NIHR Surgical Reconstruction and Microbiology Research Centre (SRMRC) and British Medical Association. The views expressed are those of the authors and not necessarily those of the NHS, the NIHR or the Department of Health.

## Author Contributions

AB, MG, VDP, DH are the principal and co- investigators.

AB, MG, VDP, DJD, ZS, MF, JRB developed the protocol.

AB, MG, VDP, DJD, ZS, MF, JRB, VS, CB drafted the protocol and commented on the protocol.

KMY, ET, ZS, CB, MF, CNW, LC, AKB drafted and finalised the manuscript.

All authors have approved the final manuscript.

## Funding

This work is supported by funding received from the NIHR Surgical Reconstruction and Microbiology Research Centre (SRMRC) and British Medical Association's Joan Dawkins Grant.

## Competing interests

The University of Birmingham owns intellectual property for some of the biomarkers used in this study.

## Patient consent

Not required.

## Ethics approval

This study was approved by the East of England - Essex Research Ethics Committee on 22 September 2017 – REC 17/EE/0275; IRAS 216703.

ISRCTN, ISRCTN16974791. Registered on 16/05/2018.

For peer review only

# References

1- McCrory P, Meeuwisse WH, Aubry M, et al. Consensus statement on concussion in sport: the 4th International Conference on Concussion in Sport held in Zurich, November 2012. Journal of the American College of Surgeons 2013;216(5):e55-71.

2- McCrory P, Meeuwisse W, Dvorak J, et al. Consensus statement on concussion in sport—the 5th international conference on concussion in sport held in Berlin, October 2016. Br J Sports Med 2017;51:838-847.

3- Langlois JA, Rutland-Brown W, Wald MM. The epidemiology and impact of traumatic brain injury: a brief overview. J Head Trauma Rehabil 2006;21: 375–378.

4- Broglio SP, Collins MW, Williams RM, et al. Current and emerging rehabilitation for concussion: a review of the evidence. Clinics in sports medicine 2015;34(2):213-31.

5- Heitger MH, Jones RD, Macleod AD, et al. Impaired eye movements in post-concussion syndrome indicate suboptimal brain function beyond the influence of depression, malingering or intellectual ability. Brain 2009;132(10):2850-70.

6- Prins ML, Alexander D, Giza CC, et al. Repeated mild traumatic brain injury: mechanisms of cerebral vulnerability. Journal of neurotrauma 2013;30(1):30-8.

7- Prins ML, Hales A, Reger M, et al. Repeat traumatic brain injury in the juvenile rat is associated with increased axonal injury and cognitive impairments. Dev Neurosci 2010;32(5-6):510-8.

8- Shrey DW, Griesbach GS, Giza CC. The pathophysiology of concussions in youth. Phys Med Rehabil Clin N Am 2011;22(4):577-602,vii.

9- Lehman EJ, Hein MJ, Baron SL, et al. Neurodegenerative causes of death among retired National Football League players. Neurology 2012;79(19):1970-4.

10- Hart J, Kraut MA, Womack KB, et al. Neuroimaging of cognitive dysfunction and depression in aging retired National Football League players: a cross-sectional study. JAMA Neurology 2013;70(3):326-35.

For peer review only

11- Gavett BE, Stern RA, McKee AC. Chronic traumatic encephalopathy: a potential late effect of sport-related concussive and subconcussive head trauma. Clinics in sports medicine 2011;30(1):179-88,xi.

12- Eyres S, Carey A, Gilworth G, et al. Construct validity and reliability of the Rivermead Post-Concussion Symptoms Questionnaire. Clinical Rehabilitation 2005;19(8):878-87.

13- Maddocks DL, Dicker GD, Saling MM. The assessment of orientation following concussion in athletes. Clin J Sport Med. 1995;5(1):32-5.

14- The National Institute for Health and Care Excellence (NICE). Head injury: assessment and early management. 2017 https://www.nice.org.uk/guidance/cg176 (accessed 14 February 2019).

15- West TA, Marion DW. Current recommendations for the diagnosis and treatment of concussion in sport: a comparison of three new guidelines. Journal of neurotrauma 2014;31(2), pp.159-168.

16- Arnett PA, Rabinowitz AR, Vargas GA, et al. Neuropsychological testing in sports concussion management: An evidence-based model when baseline is unavailable. Concussions in athletics 2014 (pp. 35-48). Springer, New York, NY.

17- Alsalaheen B, Stockdale K, Pechumer D, et al. Measurement error in the immediate postconcussion assessment and cognitive testing (ImPACT): systematic review. The Journal of head trauma rehabilitation 2016;31(4), pp.242-251.

18- Merz ZC, Roskos PT, Gfeller JD, et al. Impact of psychiatric symptomatology on neuropsychological assessment performance in persons with TBI: a comparison of OEF/OIF veteran and civilian samples. Brain injury 2017; 31(11), pp.1422-1428.

19- Resch JE, Macciocchi S, Ferrara MS. Preliminary evidence of equivalence of alternate forms of the ImPACT. Clin Neuropsychol 2013;27(8):1265-80.

20- Temple RO, Zgaljardic DJ, Abreu BC, et al. Ecological validity of the neuropsychological assessment battery screening module in post-acute brain injury rehabilitation. Brain Injury 2009;23(1):45-50.

For peer review only

21- Yungher D, Craelius W. Improving fine motor function after brain injury using gesture recognition biofeedback. Disabil Rehabil Assist Technol 2012;7(6):464-8.

22- Green P, Montijo J, Brockhaus R. High specificity of the Word Memory Test and Medical Symptom Validity Test in groups with severe verbal memory impairment. Appl Neuropsychol 2011;18(2):86-94.

23- Sollman MJ, Berry DT. Detection of inadequate effort on neuropsychological testing: a meta-analytic update and extension. Arch Clin Neuropsychol 2011;26(8):774-89.

24- Guskiewicz KM, Ross SE, Marshall SW. Postural Stability and Neuropsychological Deficits After Concussion in Collegiate Athletes. J Athl Train 2001;36(3):263-73.

25- Teel EF, Slobounov SM. Validation of a virtual reality balance module for use in clinical concussion assessment and management. Clin J Sport Med 2015;25(2):144-8.

26- Powers KC, Kalmar JM, Cinelli ME. Recovery of static stability following a concussion. Gait Posture 2014;39(1):611-4.

27- Echemendia RJ, Meeuwisse W, McCrory P, et al. The Sport Concussion Assessment Tool 5th Edition (SCAT5): Background and rationale. Br J Sports Med 2017;51:848-850.

28- Ainsworth BE, Haskell WL, Whitt MC, et al. Compendium of physical activities: an update of activity codes and MET intensities. Med Sci Sports Exerc 2000;32(9 Suppl):S498-504.

29- Vagnozzi R, Signoretti S, Cristofori L, et al. Assessment of metabolic brain damage and recovery following mild traumatic brain injury: a multicentre, proton magnetic resonance spectroscopic study in concussed patients. Brain 2010;133(11):3232-42.

30- Mariappan YK, Glaser KJ, Ehman RL. Magnetic resonance elastography: a review. Clinical Anatomy 2010;23(5):497-511.

31- Low G, Kruse SA, Lomas DJ. General review of magnetic resonance elastography. World J Radiol 2016;8(1):59-72.

For peer review only

32- Forcione M, Colonnese C, Belli A. Cerebral Hemodynamic Influences in Task-Related Functional Magnetic Resonance Imaging and Near-Infrared Spectroscopy in Acute Sport-Related Concussion: A Review. Journal of Imaging 2018;4(4),59.

33- Dehghani H, Eames ME, Yalavarthy PK, et al. Near infrared optical tomography using NIRFAST: Algorithm for numerical model and image reconstruction. Communications in numerical methods in engineering 2008;25(6),711-732.

For peer review only

# An investigation into Repetitive Concussion in Sport (RECOS): A study protocol of a prospective, exploratory, observational cohort study


| | |
|---|---|
| Journal: | *BMJ Open* |
| Manuscript ID | bmjopen-2019-029883.R1 |
| Article Type: | Protocol |
| Date Submitted by the Author: | 24-May-2019 |
| Complete List of Authors: | Yakoub, Kamal; NIHR SRMRC, Trauma<br>Davies, David; NIHR Surgical Reconstruction and Microbiology Research Centre, University Hospitals Birmingham NHS Foundation Trust<br>Su, Zhangjie; NIHR SRMRC<br>Bentley, Conor; NIHR Surgical Reconstruction and Microbiology Research Centre, University Hospitals Birmingham NHS Foundation Trust<br>Forcione, Mario; NIHR Surgical Reconstruction and Microbiology Research Centre, University Hospitals Birmingham NHS Foundation Trust,<br>Toman, Emma; NIHR Surgical Reconstruction and Microbiology Research Centre, Neurosurgery<br>Hammond, Douglas; Head Injury Management Research Group, Faculty of Clinical and Biomedical Science, School of Dentistry, University of Central Lancashire,<br>Watson, Callum; Neurotrauma and Ophthalmology Research Group, Institute of Inflammation and Aging, University of Birmingham,<br>Bishop, Jon; NIHR Surgical Reconstruction and Microbiology Research Centre, Medical Statistician<br>Cooper, Lauren; NIHR Surgical Reconstruction and Microbiology Research Centre, University Hospitals Birmingham NHS Foundation Trust Queen Elizabeth Hospital Birmingham<br>Barbey, Aron; The Beckman Institute for Advanced Science and Technology, University of Illinois<br>Sawlani, Vijay; University Hospitals Birmingham NHS Foundation Trust, Department of Radiology, Queen Elizabeth Hospital,<br>Di Pietro, V; University of Birmingham,<br>Grey, Michael<br>belli, antonio; University of Birmingham, Institute of Inflammation and Ageing |
| <b>Primary Subject Heading</b>: | Sports and exercise medicine |
| Secondary Subject Heading: | Diagnostics, Neurology, Radiology and imaging |
| Keywords: | Concussion, Traumatic brain injury, Sport concussion, Biomarkers, Imaging, Post-concussion syndrome |
| | |


For peer review only - http://bmjopen.bmj.com/site/about/guidelines.xhtml

# Title

An investigation into Repetitive Concussion in Sport (RECOS): A study protocol of a prospective, exploratory, observational cohort study

# Authors

Kamal M Yakoub[1,2], David J Davies[1,2], Zhangjie Su[1], Conor Bentley[1,2], Mario Forcione[1,2] , Emma Toman[3], Douglas Hammond[3], Callum N Watson[2], Jon Bishop[1], Lauren Cooper[1], Aron K Barbey[6], Vijay Sawlani[1,4], Valentina Di Pietro[1,2,6], Michael J Grey[5], Antonio Belli[1,2]

# Correspondence to

Dr Kamal M Yakoub

NIHR Surgical Reconstruction and Microbiology Research Centre

University Hospitals Birmingham NHS Foundation Trust

4th Floor West, Institute of Translational Medicine

Heritage Building (Queen Elizabeth Hospital)

B15 2TH

Birmingham

United Kingdom

E-mail: k.yakoub@bham.ac.uk

Telephone: +44(0)1213718223

# Author affiliations

1 NIHR Surgical Reconstruction and Microbiology Research Centre, University Hospitals Birmingham NHS Foundation Trust, Birmingham, UK

2 Neurotrauma and Ophthalmology Research Group, Institute of Inflammation and Ageing, University of Birmingham, Birmingham, UK

3 Head Injury Management Research Group, Faculty of Clinical and Biomedical Science, School of Dentistry, University of Central Lancashire, Preston, UK

4 Department of Neuroradiology, Queen Elizabeth Hospital Birmingham, University Hospitals Birmingham NHS Foundation Trust, Birmingham, UK

5 Acquired Brain Injury Rehabilitation Alliance (ABIRA), School of Health Sciences, University of East Anglia, Norwich, UK

6 The Beckman Institute for Advanced Science and Technology, University of Illinois at Urbana Champaign, Illinois, USA



# Keywords

Concussion; Traumatic brain injury; Sport concussion; Biomarkers; Imaging; Post-concussion syndrome

# Abstract

## Introduction

Sport-related concussion (SRC) management remains a diagnostic dilemma to clinicians in all strata of care, coaching staff and players alike. The lack of objective diagnostic and prognostic biomarkers and over-reliance on subjective clinical assessments carries a significant health risk of undiagnosed concussive episodes and early return to play before full recovery increasing the risk of sustaining additional concussion, and leading to long-term sequelae and/or unfavorable outcome.

## Objective

To identify a set of parameters (neuroimaging with neurophysiological, biological and neuropsychological tests) that may support pitch-side and outpatient clinical decision making in order to objectively diagnose concussion, determine the severity of injury, guide a safe return to play and identify the potential predictors of the long-term sequelae of concussion.

## Methods and analysis

An exploratory, observational, prospective, cohort study recruiting between 2017 and 2020.

The participants will have a baseline pre-season screening (brain imaging, neuropsychological assessments, serum, urine and saliva sampling). If a screened player later suffers a concussion and/or multiple concussions then he/she will be assessed again with the same protocol within 72h, and their baseline data will be used as internal control as well as normative data. Inferential statistical analysis




For peer review only - http://bmjopen.bmj.com/site/about/guidelines.xhtml

will be performed to determine correlations between biological, imaging techniques and neuropsychological assessments.

**Ethics and dissemination**

This study was approved by the East of England - Essex Research Ethics Committee on 22 September 2017 – REC 17/EE/0275; IRAS 216703.

The results of this study will be presented at national and international conferences and submitted for publication in peer reviewed journals.

# Article summary

**Strengths and limitations of this study**

- Prospectively recruiting from across the contact sporting continuum, this study will collect baseline and post-concussion biological samples with corresponding neuropsychological, neurophysiological and neuroimaging tests.
- The study will help to establish a multidisciplinary approach to objectively diagnose sport-related concussion and guide a safe return to play following single and repetitive concussions.
- To inter-correlate neuroimaging with neurophysiological, biological and neuropsychological tests for the prospective development of potential pitch-side and ambulatory technologies.
- This study will recruit from a population of adult athletes and therefore applications of study findings to other populations, such as non-athletes, will require further validation.
- Neuroimaging may not be suitable for all the participants, such as pregnant, claustrophobic athletes, and those with metallic implants.



For peer review only - http://bmjopen.bmj.com/site/about/guidelines.xhtml

# Introduction

Sport-related concussion (SRC) is defined as a brief period of loss of consciousness (LOC), memory loss, feeling dazed or confused following trauma to the head, face or neck, and is a common cause of mild traumatic brain injury (mTBI).[1,2] In the US, the Centers for Disease Control and Prevention estimate that 1.6 to 3.8 million concussions occur in sports and recreational activities annually, but there is fear that the number may be much larger, as the majority of incidents are unrecognized or unreported.[3]

The majority of patients improve rapidly following a single concussion, but 10-20% of individuals have persistent symptoms (e.g. headache, dizziness, fogginess, imbalance, and anxiety) at three months. In a minority (2-4%) the symptoms may become permanent. [4,5] There is a higher risk of sustaining further concussion if they returned to play before full recovery which prolongs recovery time after each incident.[4, 5]

Certain groups of patients, such as athletes, soldiers and children are at greater risk of repetitive mild traumatic brain injury (mTBI); potentially leading to a catastrophic form of brain injury known as second impact syndrome (SIS), thought to be due to the second insult occurring during a window of metabolic vulnerability of the brain.[6-8]

In susceptible individuals, repetitive head trauma has been linked to early neurodegenerative conditions such as Parkinson's, amyotrophic lateral sclerosis and Alzheimer's disease. The risk of neurodegenerative conditions has been demonstrated to treble in retired professional American football players [9] and following the media attention dedicated to this issue, the link between mTBI and chronic traumatic encephalopathy (CTE) has become a major public concern. [10, 11]



For peer review only - http://bmjopen.bmj.com/site/about/guidelines.xhtml

Compounding this problem is the fact that athletes, like soldiers, are highly trained, young, fit, motivated individuals proven to under-report concussive symptoms.[12, 13]

Athletes are, therefore, at risk of SIS and CTE. Currently, there is no validated clinical biomarker available to assess mTBI, SIS and cumulative brain damage, leading to an over-reliance on self-reporting of symptoms in the management of mTBI.[14]

At present, the mainstream assessment of mTBI both in sports and in the primary/secondary healthcare setting involves the functional and symptomatic assessment of an individual using neurocognitive tests.[15] These tests have significant limitations, particularly the lack of baseline/premorbid measurement in terms of sensitivity and specificity [16, 17] and the susceptibility to multiple confounding factors [18] such as musculoskeletal injury and/or pre-morbid disability. This together with their requirement for a high level of engagement and concentration limits their application in outpatient clinic or pitch-side.

## Methods and analysis

### Study design

A cohort of contact sport athletes (e.g. rugby, football, American football, etc.) will be recruited throughout the West Midlands region and through referrals from sports clubs anywhere in the Great Britain.

The assessments are carried out in two settings, (1) the sports clubs with clinical areas dedicated to sample taking and data collection and (2) Birmingham concussion clinic at the Queen Elizabeth Hospital Birmingham (QEHB) or at the University of Birmingham (UoB).



For peer review only - http://bmjopen.bmj.com/site/about/guidelines.xhtml

Eligible individuals will be identified by key members of their coaching staff who have been trained regarding the inclusion specifications and the procedures involved in recruitment (Table 1). Athletes who sustain a head injury during competition or training will be advised to attend an assessment session between 48h and 72h after their concussion at the concussion clinic at QEHB, although attending directly or as soon as is practically possible will be advised. Should the injury be sufficiently serious that the attending staff believes immediate medical assistance or transfer to the nearest emergency department is required, then standard clinical procedures should be followed in these cases.

Consent to participate in the study will be obtained from the participants in a standardised written consent form. Details of the consent will be maintained as per the UoB and University Hospitals Birmingham NHS Foundation Trust (UHBNFT) guidelines, and maintained for the study period and archived thereafter.

Consented non-concussed athletes will participate in a baseline screening consisting of same assessments as for the concussed players, although these may not include Magnetic Resonance Imaging (MRI) screening in all subjects due to resources constrains. The uninjured cohort provides normative data, as well as internal control data if a screened player later suffers a concussion during the season.  Pre-season screening assessments will be conducted at either the University of Birmingham site or the QEHB site or at the club facilities.



For peer review only - http://bmjopen.bmj.com/site/about/guidelines.xhtml

Up to 400 singly or multiply concussed athletes (where the second concussion has occurred within 21 days of the first event) will be recruited, consented and assessed by:

- Sampling of 25ml venous blood, 2ml saliva and 5ml urine for metabolomics and genomic analysis (Table 2).
- The Immediate post-concussion assessment and cognitive testing (ImPACT) is a computerised neurocognitive test for evaluating sport-related concussion. It measures multiple aspects of cognitive functioning, and consists of five testing composites, i.e. verbal memory, visual memory, processing speed, reaction time, and impulse control.[19]
- The Screening Module of Neuropsychological Assessment Battery (NAB) (Digit Span).[18]
- Wechsler Adult Intelligence Scale Version 4 (WAIS-IV) (Digit Symbol Coding and Symbol Search).[20]
- Nine Hole Peg Test (NHPT) to assess fine motor skills; the individual will be asked to place and remove nine pegs at one time, as quickly as possible, from nine holes in a peg board. This test evaluates coordination of upper limbs and hand dexterity. [21]
- The Medical Symptom Validity Test (MSVT) is a validated and computerized performance validity test. It is designed to assess the degree to which the participant has engaged appropriately in the testing process. [22]
- Balance assessments including virtual reality system, gait analysis, Modified Balance Error Scoring System (mBESS). [20-27]

  The mBESS test will be performed whereby a trained member of the RECOS team will assess the number of errors participants make whilst

For peer review only



For peer review only - http://bmjopen.bmj.com/site/about/guidelines.xhtml

undertaking eyes closed dual, single leg and a tandem stances for 30 seconds respectively.[27]

- An Immersion questionnaire to complete after the virtual reality system to investigate their level of immersion and sense of presence in the virtual reality environment and questionnaire using the Metabolic Equivalent of Tasks (MET) will be used to assess participants' physical activity levels prior to the sustaining of a concussion.[28]
- MRI to assess neuronal energy metabolism, functional brain network and tissue mechanical parameters.
- Functional Near-Infrared Spectroscopy (fNIRS) to assess brain activation patterns during neurocognitive tasks.

The Sport Concussion Assessment Tool – 5th Edition (SCAT5)[27] may be taken within the sport club facilities immediately after concussion. Any other data will be collected during the pitch-side clinical assessment on concussed athletes within the Rugby Football Union guidelines, i.e. the Injury Surveillance Programme, may be used in the study. After the match the concussed participants will visit QEHB or the UoB or dedicated sport club facilities where all the assessments will be completed with short breaks in-between when necessary (figure 1).

Magnetic Resonance (MR) scanning (approximately 1h) will be performed using a 3T MR scanner at the QEHB. First of all, a standard structural MR scan will be acquired (T1-, T2-, and T2*-weighted and FLAIR images), followed by MR Spectroscopy (1H-MRS) (NAA/Choline, NAA/Creatine and Choline/Creatine ratios.)[29], resting-state functional MRI (fMRI) (subjects are awake, eyes closed, motionless), Diffusion tensor imaging (DTI), and Magnetic Resonance Elastography (MRE) on brain will be performed to obtain quantitative values for tissue mechanical parameters.[30, 31]



For peer review only - http://bmjopen.bmj.com/site/about/guidelines.xhtml

Prior to any female participant undergoing MR imaging, a pregnancy test will be performed after the expressed consent of the individual.

These assessments will be repeated in the QEHB or sports facilities during the follow up and when the individual is deemed fit to return to play, until normalisation of the MR Spectroscopy (1H-MRS) or self-reported symptoms are observed.

The Near-Infrared Spectroscopy (NIRS) is a safe non-invasive brain imaging technique that utilises Near-Infrared (NIR) light absorption to assess brain oxygen saturation. The NIR light is currently widely used in clinical practice (e.g. pulse oximeter and brain monitoring during cardiac surgery). fNIRS measurements are performed once within 48-72 hours from the injury using a frequency domain NIRS device (ISS™ IMAGENT; Champaign, Illinois, USA) and a digitizer (POLHEMUS FASTRAK®; Colchester, Vermont, USA).[32,33]



For peer review only - http://bmjopen.bmj.com/site/about/guidelines.xhtml

## Outcome measures and statistical analysis

The recruitment target is based on the size of the convenient sample that can be recruited (approximately 400); power calculation do not apply as the investigation is explorative at this stage. The sample size will be open-ended and reviewed after 3 years.

Correlations between biomarkers, 1H-MRS, fMRI, MRE, DTI, fNIRS, neuropsychological scores, motor and coordination parameters will be assessed and reported with standard parametric statistical tests after normalisation of data or non-parametric test if normalisation were not possible. Time to resolution of symptoms will be compared for all the above modalities, as well as self-reported outcomes. Biochemical and imaging data will also be analysed with standard group comparison statistics (e.g. t-test on normalised data or Mann-Whitney on non-normalisable data).

## Patient and Public Involvement

Links with sports academics and coaching staff have been established throughout the region through the University of Birmingham School of Sports, Exercise and Rehabilitation Sciences (SportexR) and through sports authorities such as the Rugby Football Union, the Welsh Rugby Union, GB Basketball, GB Hockey and the Football Association. Awareness of these programs will be promoted to the potential cohort of athletes through event days organised at individual recruitment sites coordinated centrally through the UoB. In terms of dissemination, the study team will regularly update the clubs on the progress of the study and will report the findings by email/newsletter and in various meetings where club representatives are in attendance.



For peer review only - http://bmjopen.bmj.com/site/about/guidelines.xhtml

# Ethics and dissemination

This study was approved by the East of England - Essex Research Ethics Committee on 22 September 2017 – REC 17/EE/0275; IRAS 216703.

ISRCTN, ISRCTN16974791. Registered on 16/05/2018.

All data will be collected and stored in accordance with the 1998 UK Data Protection Act, UoB and QEHB data handling and maintenance guidelines, with the minimum amount of required information recorded. The complied and analysed results will be presented at national and international conferences. Results will also be submitted for peer review and publication in the subject journals/literature.

## Data statement

All analysed data arising from the study will be kept on NHS and UoB servers. The computers on this network have restricted physical access; data are stored under coded filenames and the local network has secure password access restricted to a limited set of people. The raw MRI data are kept on a separate DICOM server on another network behind its own firewall which is only accessible by a small number of system administrators. Data spreadsheets will be kept on computers with password protected access and coded filenames, the original paper files will be secured in locked filing cabinets.

The datasets generated and/or analysed during the current study are/will be available upon request from, Professor Antonio Belli, who is the point of contact and will be able to provide anonymised data with sufficient details to able to reproduce the analyses for up to 10 years.



For peer review only - http://bmjopen.bmj.com/site/about/guidelines.xhtml

## Acknowledgements and Disclaimer

The authors acknowledge UoB, NIHR SRMRC and British Medical Association for their support. Also, we would like to thank all the sports authorities, clubs and academics that agreed to participate in the study. This study is funded by the National Institute for Health Research (NIHR) Surgical Reconstruction and Microbiology Research Centre (SRMRC). The views expressed are those of the authors and not necessarily those of the NIHR or the Department of Health and social care.

## Author Contributions

AB, MJG, VDP, DH are the principal and co- investigators.
AB, MJG, VDP, DJD, ZS, MF, JB developed the protocol.
AB, MJG, VDP, DJD, ZS, MF, JB, VS, CB drafted the protocol and commented on the protocol.
KMY, ET, ZS, CB, MF, CNW, LC, AKB drafted and finalised the manuscript.
All authors have approved the final manuscript.

## Funding

This work is supported by funding received from the National Institute for Health Research (NIHR) Surgical Reconstruction and Microbiology Research Centre (SRMRC).

## Competing interests

The University of Birmingham owns intellectual property for some of the biomarkers used in this study.

## Patient consent

Not required.

## Ethics approval

This study was approved by the East of England - Essex Research Ethics Committee on 22 September 2017 – REC 17/EE/0275; IRAS 216703.
ISRCTN, ISRCTN16974791. Registered on 16/05/2018.

For peer review only

# References


1- McCrory P, Meeuwisse WH, Aubry M, et al. Consensus statement on concussion in sport: the 4th International Conference on Concussion in Sport held in Zurich, November 2012. Journal of the American College of Surgeons 2013;216(5):e55-71.

2- McCrory P, Meeuwisse W, Dvorak J, et al. Consensus statement on concussion in sport—the 5th international conference on concussion in sport held in Berlin, October 2016. Br J Sports Med 2017;51:838-847.

3- Langlois JA, Rutland-Brown W, Wald MM. The epidemiology and impact of traumatic brain injury: a brief overview. J Head Trauma Rehabil 2006;21: 375–378.

4- Broglio SP, Collins MW, Williams RM, et al. Current and emerging rehabilitation for concussion: a review of the evidence. Clinics in sports medicine 2015;34(2):213-31.

5- Heitger MH, Jones RD, Macleod AD, et al. Impaired eye movements in post-concussion syndrome indicate suboptimal brain function beyond the influence of depression, malingering or intellectual ability. Brain 2009;132(10):2850-70.

6- Prins ML, Alexander D, Giza CC, et al. Repeated mild traumatic brain injury: mechanisms of cerebral vulnerability. Journal of neurotrauma 2013;30(1):30-8.

7- Prins ML, Hales A, Reger M, et al. Repeat traumatic brain injury in the juvenile rat is associated with increased axonal injury and cognitive impairments. Dev Neurosci 2010;32(5-6):510-8.

8- Shrey DW, Griesbach GS, Giza CC. The pathophysiology of concussions in youth. Phys Med Rehabil Clin N Am 2011;22(4):577-602,vii.

9- Lehman EJ, Hein MJ, Baron SL, et al. Neurodegenerative causes of death among retired National Football League players. Neurology 2012;79(19):1970-4.

10- Hart J, Kraut MA, Womack KB, et al. Neuroimaging of cognitive dysfunction and depression in aging retired National Football League players: a cross-sectional study. JAMA Neurology 2013;70(3):326-35.




For peer review only - http://bmjopen.bmj.com/site/about/guidelines.xhtml

11- Gavett BE, Stern RA, McKee AC. Chronic traumatic encephalopathy: a potential late effect of sport-related concussive and subconcussive head trauma. Clinics in sports medicine 2011;30(1):179-88,xi.

12- Eyres S, Carey A, Gilworth G, et al. Construct validity and reliability of the Rivermead Post-Concussion Symptoms Questionnaire. Clinical Rehabilitation 2005;19(8):878-87.

13- Maddocks DL, Dicker GD, Saling MM. The assessment of orientation following concussion in athletes. Clin J Sport Med. 1995;5(1):32-5.

14- The National Institute for Health and Care Excellence (NICE). Head injury: assessment and early management. 2017 https://www.nice.org.uk/guidance/cg176 (accessed 14 February 2019).

15- West TA, Marion DW. Current recommendations for the diagnosis and treatment of concussion in sport: a comparison of three new guidelines. Journal of neurotrauma 2014;31(2), pp.159-168.

16- Arnett PA, Rabinowitz AR, Vargas GA, et al. Neuropsychological testing in sports concussion management: An evidence-based model when baseline is unavailable. Concussions in athletics 2014 (pp. 35-48). Springer, New York, NY.

17- Alsalaheen B, Stockdale K, Pechumer D, et al. Measurement error in the immediate postconcussion assessment and cognitive testing (ImPACT): systematic review. The Journal of head trauma rehabilitation 2016;31(4), pp.242-251.

18- Merz ZC, Roskos PT, Gfeller JD, et al. Impact of psychiatric symptomatology on neuropsychological assessment performance in persons with TBI: a comparison of OEF/OIF veteran and civilian samples. Brain injury 2017; 31(11), pp.1422-1428.

19- Resch JE, Macciocchi S, Ferrara MS. Preliminary evidence of equivalence of alternate forms of the ImPACT. Clin Neuropsychol 2013;27(8):1265-80.

20- Temple RO, Zgaljardic DJ, Abreu BC, et al. Ecological validity of the neuropsychological assessment battery screening module in post-acute brain injury rehabilitation. Brain Injury 2009;23(1):45-50.



For peer review only - http://bmjopen.bmj.com/site/about/guidelines.xhtml

375 21- Yungher D, Craelius W. Improving fine motor function after brain injury using gesture recognition biofeedback. Disabil Rehabil Assist Technol 2012;7(6):464-8.

377 22- Green P, Montijo J, Brockhaus R. High specificity of the Word Memory Test and Medical Symptom Validity Test in groups with severe verbal memory impairment. Appl Neuropsychol 2011;18(2):86-94.

380 23- Sollman MJ, Berry DT. Detection of inadequate effort on neuropsychological testing: a meta-analytic update and extension. Arch Clin Neuropsychol 2011;26(8):774-89.

382 24- Guskiewicz KM, Ross SE, Marshall SW. Postural Stability and Neuropsychological Deficits After Concussion in Collegiate Athletes. J Athl Train 2001;36(3):263-73.

384 25- Teel EF, Slobounov SM. Validation of a virtual reality balance module for use in clinical concussion assessment and management. Clin J Sport Med 2015;25(2):144-8.

386 26- Powers KC, Kalmar JM, Cinelli ME. Recovery of static stability following a concussion. Gait Posture 2014;39(1):611-4.

388 27- Echemendia RJ, Meeuwisse W, McCrory P, et al. The Sport Concussion Assessment Tool 5th Edition (SCAT5): Background and rationale. Br J Sports Med 2017;51:848-850.

390 28- Ainsworth BE, Haskell WL, Whitt MC, et al. Compendium of physical activities: an update of activity codes and MET intensities. Med Sci Sports Exerc 2000;32(9 Suppl):S498-504.

392 29- Vagnozzi R, Signoretti S, Cristofori L, et al. Assessment of metabolic brain damage and recovery following mild traumatic brain injury: a multicentre, proton magnetic resonance spectroscopic study in concussed patients. Brain 2010;133(11):3232-42.

395 30- Mariappan YK, Glaser KJ, Ehman RL. Magnetic resonance elastography: a review. Clinical Anatomy 2010;23(5):497-511.

397 31- Low G, Kruse SA, Lomas DJ. General review of magnetic resonance elastography. World J Radiol 2016;8(1):59-72.



For peer review only - http://bmjopen.bmj.com/site/about/guidelines.xhtml

32- Forcione M, Colonnese C, Belli A. Cerebral Hemodynamic Influences in Task-Related Functional Magnetic Resonance Imaging and Near-Infrared Spectroscopy in Acute Sport-Related Concussion: A Review. Journal of Imaging 2018;4(4),59.

33- Dehghani H, Eames ME, Yalavarthy PK, et al. Near infrared optical tomography using NIRFAST: Algorithm for numerical model and image reconstruction. Communications in numerical methods in engineering 2008;25(6),711-732.

For peer review only



For peer review only - http://bmjopen.bmj.com/site/about/guidelines.xhtml

# Figure legends/caption

Table 1

Summary of eligibility criteria

| Inclusion criteria | Exclusion criteria |
|---|---|
| Male or female athletes participating in contact sports, aged 16-65 years, with fluent English speaking. | Individuals who require hospital admission after initial assessment for their TBI. |
| Single or repetitive mTBI sustained in contact sport less than 72 hours prior to assessment. | Intracranial haemorrhage, brain tissue injury, or non-TBI related pathologies on initial CT/MRI scan. |
| Normal neurological objective examination at the time of enrolment. | Pregnancy (urine pregnancy test will be performed for confirmation). |
| | Any history of neurodegenerative pathology or any recent or ongoing illness affecting the central nervous system (e.g. Parkinson's, multiple sclerosis, meningitis, epilepsy, neoplasm). |
| | History of chronic alcoholism or drug abuse. |
| | Any other sustained injury that requires hospital admission. |

For peer review only



For peer review only - http://bmjopen.bmj.com/site/about/guidelines.xhtml

Table 2

Analysis of biological fluids

| Blood | Urine | Saliva |
|---|---|---|
| 1. Metabolomics (NAA and related metabolites)<br>2. Hormones (Progesterone, Aldosterone,11-deoxycortisol, Corticosterone, Testosterone, Androstenedione, Cortisol, 17OHP, DHEA, DHEAS, Cortisone)<br>3. Pro and anti-inflammatory cytokines (TNFα, IL1β, IL6, IL4, IL8, IL10, IL13, IL17, GM-CSF)<br>4. Proteomics<br>5. Microparticles<br>6. Biomarkers of brain injury (S100B, GFAP and NSE)<br>7. MicroRNAs | 1. Hormone profile<br>2. Brain biomarkers (NAA, S100B and GFAP)<br>3. Metabolomics<br>4. MicroRNAs | 1. MicroRNAs<br>2. Metabolomics |

Figure 1

Flowchart of study procedures

***urine pregnancy test will be performed first for all female participants**



For peer review only - http://bmjopen.bmj.com/site/about/guidelines.xhtml

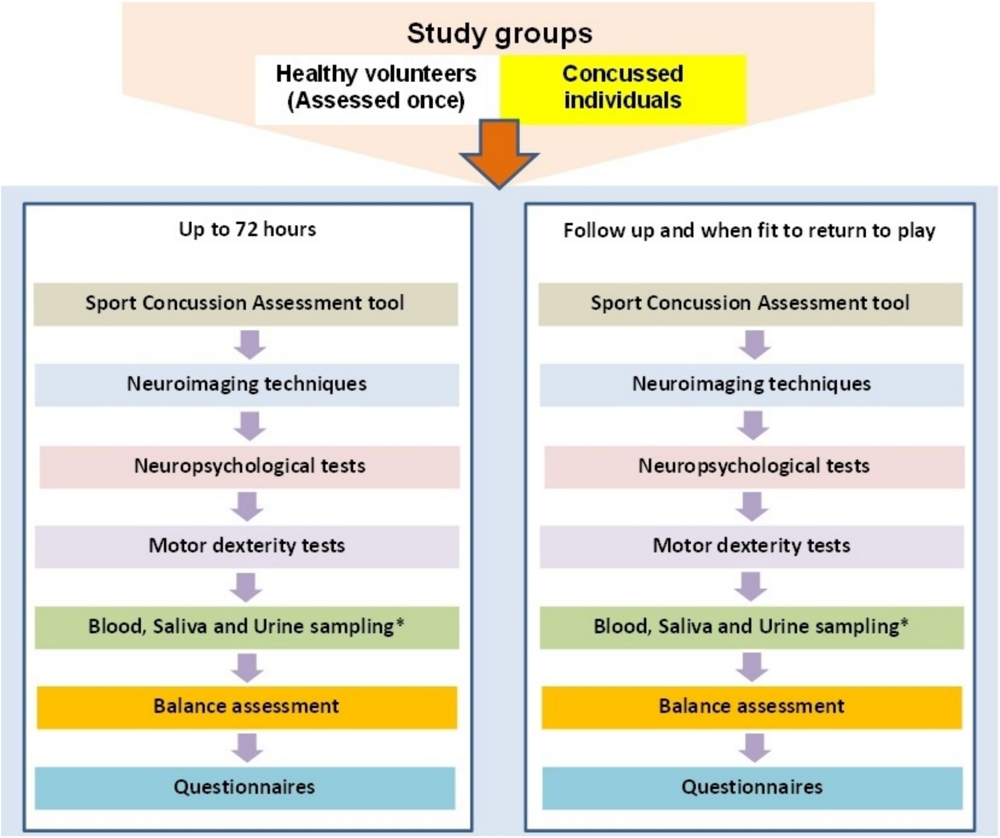


Figure 1/ Flowchart of study procedures

70x58mm (300 x 300 DPI)
