# Supplementary material for: Investigation into repetitive concussion in sport (RECOS): study protocol of a prospective, exploratory, observational cohort study
Source: BMJ Open. 2019 Jul 4;9(7):e029883. doi: 10.1136/bmjopen-2019-029883 (PMC6615833; doi:10.1136/bmjopen-2019-029883)
Supplement: Reviewer comments [file bmjopen-2019-029883.reviewer_comments.pdf]

## PEER REVIEW HISTORY

BMJ Open publishes all reviews undertaken for accepted manuscripts. Reviewers are asked to complete a checklist review form (**http://bmjopen.bmj.com/site/about/resources/checklist.pdf**) and are provided with free text boxes to elaborate on their assessment. These free text comments are reproduced below.

## ARTICLE DETAILS

| | |
|---|---|
| **TITLE (PROVISIONAL)** | An investigation into Repetitive Concussion in Sport (RECOS): A study protocol of a prospective, exploratory, observational cohort study |
| **AUTHORS** | Yakoub, Kamal; Davies, David; Su, Zhangjie; Bentley, Conor; Forcione, Mario; Toman, Emma; Hammond, Douglas; Watson, Callum; Bishop, Jon; Cooper, Lauren; Barbey, Aron; Sawlani, Vijay; Di Pietro, V; Grey, Michael; belli, antonio |

## VERSION 1 - REVIEW

| | |
|---|---|
| **REVIEWER** | Jiri Dvorak<br>Swiss Concussion Center Schulthess Clinic Zurich Switzerland |
| **REVIEW RETURNED** | 01-Apr-2019 |

| | |
|---|---|
| **GENERAL COMMENTS** | well design study based upon the CISG publications |

| | |
|---|---|
| **REVIEWER** | Allen Sills<br>Vanderbilt University Medical Center Nashville, TN USA<br>I am a full time employee of the National Football League (NFL) |
| **REVIEW RETURNED** | 18-Apr-2019 |

| | |
|---|---|
| **GENERAL COMMENTS** | Would like to have additional discussion of limitations of the research questions. |

## VERSION 1 – AUTHOR RESPONSE

Reviewer: 2

Would like to have additional discussion of limitations of the research questions.

Page 5 lines 97-101

Page 8 lines 181-183

Page 15 line 283
